# Potentiating effects of *Perovskia abrotanoides* Karel. on pentobarbital-induced sleep

**Published:** 2017

**Authors:** Fatemeh Forouzanfar, Azar Hosseini, Mohammad Sadegh Amiri, Hassan Rakhshandeh

**Affiliations:** 1 *Pharmacological Research Center of Medicinal Plants, Mashhad University of Medical Sciences, Mashhad, Iran*; 2 *Department of Biology, Payame Noor University, Tehran, Iran*; 3 *Department of Pharmacology, School of Medicine, Mashhad University of Medical Sciences, Mashhad, Iran*

**Keywords:** Perovskia abrotanoides, Diazepam, Sleep, PC12

## Abstract

**Objective:**

Sleeplessness is the most common sleep disorder. In this study the hypnotic effect of the hydro-alcoholic extract (HAE) of *Perovskia abrotanoides* Karel. and its water fraction (WF), ethyl acetate fraction (EAF) and n-butanol fraction (NBF) were studied in mice.

**Materials and Methods:**

The test compounds were administered intraperitoneally to mice 30 min before the administration of sodium pentobarbital (30 mg/kg.). Moreover, the influence of flumazenil on the hypnotic effect of the extracts was evaluated. Besides, 30 min after administration of HAE, motor coordination (rota-rod test) was assessed. Additionally, LD50 for HAE was determined and the possible neurotoxicity of the extract was tested in neural PC12 cells.

**Results:**

The HAE and NBF decreased the latency of sleep (p<0.05), and significantly increased the duration of sleep (p<0.05) induced by pentobarbital. These effects of *P. abrotanoides* were reversed by flumazenil. HAE did not affect the animals’ performance on the rota-rod test. The LD50 value for HAE was found to be 4.8 g/kg. HAE and its fractions did not show neurotoxic effects in cultured PC12 cell line.

**Conclusion:**

The results showed that *P. abrotanoides *significantly potentiated pentobarbital hypnosis without toxic effect. Probably, its effects are related to its non-polar constituents.

## Introduction

Insomnia is a common problem. Thirty to fifty percent of the populations suffer from insomnia. Insomnia may lead to physical problems including poor memory, slower reactions, emotional disturbances and changes in the immune response (Orzel-Gryglewska, 2010 ▶; Zaharna and Guilleminault, 2010 ▶). Individuals suffering from insomnia take drugs to improve their sleep status (Valli`eres et al., 2005 ▶). Common drugs that are used in sleep disorder include benzodiazepines/non-benzodiazepines, anti-depressants and anti-histamines. But, administration of these agents for long time leads to various adverse effects such as impaired cognitive function, memory and general daytime performance, tolerance and dependence (Cho et al., 2010 ▶). Therefore, there has been a growing demand for food constituents and natural products with hypnotic effects. Recent studies have shown that foods and herbal plants contain specific constituents which may be effective in improving sleep quality and avoiding side effects (Meletis, 2008 ▶). Some of these plants which have hypnotic effect are *Lactuca sativa (*Ghorbani A et al., 2013 ▶), *Lactuca serriola *(Hosseini et al., 2014 ▶), *Crocus sativus* (Hosseinzadeh et al., 2009 ▶), *Rosa damascene *(Rakhshandah and Hosseini, 2006 ▶), *Coriandrum sativum *(Rakhshandeh et al., 2011 ▶) that have beneficial effects in animal models of sleep disorder*. *Some of neurotransmitters are involved in sleeping regulation such as GABAergic neurotransmission. The most of hypnotic-sedative drugs act via GABA-A receptor (Johnston et al., 2005 ▶). Past studies have shown that polyphenols and flavonoids have sedative-hypnotic effects based on positive allosteric modulation of GABA-A receptors (Bateson, 2012 ▶). *Perovskia abrotanoides* (Lamiaceae), locally known as “hoosh”, “visk”, “brazambal”, “domou”, and “gevereh” (Mahboubi and Kazempour, 2009 ▶, Mazandarani and Ghaemi, 2010 ▶), grows in Afghanistan, Iran, Pakistan and Turkmenistan (Mozaffarian, 1998 ▶). Phytochemical screening of the aerial parts of *P. abrotanoides *showed that it has high content of monoterpenes and sesquiterpenes like 1, 8-cineolo, myrcene, pinene, camphor, caryophyllene, humulene, camphene and bisabolol (Sajadi et al., 2005 ▶; Morteza-Semnani, 2004 ▶). In traditional medicine, it is used in the treatment of typhoid, headache, gonorrhea, vomiting, motion, toothache, atherosclerosis, cardiovascular diseases, liver fibrosis, and cough (Moallem and Niapour, 2008 ▶; Kumar et al., 2009 ▶; Tareen et al., 2010 ▶). Also, it has sedative, analgesic, antiseptic and cooling effects (Moallem and Niapour, 2008 ▶; Hosseinzadeh and Amel, 2001 ▶, Nassiri et al., 2000 ▶). Herbal tea of this plant is used to cure infections and painful urination (Ballabh et al., 2008 ▶). Its pharmacological effects such as anti-plasmodial, anti-inflammatory and cytotoxic effects have been also shown (Sairafianpour et al., 2001 ▶, Esmaeilli et al., 2008 ▶, Beikmohammadi, 2012 ▶). Moreover, it was reported that its antioxidant activity enhances heart function (Moallem and Niapour, 2008 ▶, Rustaiyan et al., 2006 ▶). Since hypnotic effect of *P. abrotanoides* has been reported in ancient medicine, this study was designed to investigate the effect of *P. abrotanoides* on sleep.

## Materials and Methods

**Drugs and chemicals**

Pentobarbital sodium, penicillin-streptomycin and flumazenil were bought from Sigma (USA). Diazepam was purchased from Chemidarou Company (Iran). Dulbecco’s Modified Eagle’s Medium (DMEM) and fetal bovine serum (FBS) were obtained from GIBCO (USA).

**Plant Collection and extraction**

*P. abrotanoides *Karel was collected from the Tirgan mountains, Hezarmasjed protected area, Razavi Khorasan Province, Iran. A voucher specimen (No. 355 by Amiri) was prepared and deposited in the herbarium of Department of Biology, Payame Noor University, Dargaz, Iran.

The aerial parts of *P. abrotanoides *were powdered and then extracted with maceration method as 100 g of the powder was extracted using 1000 ml of 70% ethanol for 48 hr, ﬁltered and dried on a water bath. The yield of the dried extract was 40g.

To prepare different fractions of the extract, 10 g of dried hydro-alcoholic extract (HAE) was suspended in 200 ml distilled water and transferred to a separator funnel. By using solvent-solvent extraction method, ethyl acetate and n-butanol fractions were prepared. The ethyl acetate fraction (EAF) and n-butanol fraction (NBF) were separated to get water fraction (WF). All the fractions were dried on a water bath and working solutions made in saline that contains 1% tween and 10% DMSO for WF, NBF and EAF (Rakhshandeh et al., 2012 ▶). The yield of WF, NBF and EAF were 63.5%, 22.5% and 14%, respectively.

**Animals**

Male albino mice (weighing 20-30 g) and male Wistar rats (weighing 200-250 g) were kept at controlled temperature (22-24°C) with a 12 hr/12 hr light/dark cycle and had free access to water and food. The study was done in accordance with ethical rule of Mashhad University of Medical Sciences, Mashhad, Iran. The animals were randomly divided into 12 groups of 8 mice. First, hypnotic effect of HEA was evaluated in six groups: saline as negative control, diazepam (3 mg/kg) as positive control and HAE (50, 100, 150 and 200 mg/kg). 

Second, for determination of the most effective fraction, in three groups of animals, hypnotic effect of HAE fractions including WF (100 mg/kg), EAF (100 mg/kg) and NBF (50 and 100 mg/kg) were studied. Furthermore, flumazenil (2 mg/kg) was used as a benzodiazepine receptor antagonist for identifying the underlying mechanism.

**Evaluation of pentobarbital-induced sleep**

Hypnosis was evaluated based on prolongation of sleep induced by pentobarbital (Hosseini et al., 2014 ▶). Briefly, a single dose of HAE (50, 100, 150 and 200 mg/kg), fractions of HAE, diazepam (3 mg/kg), or saline was administered intraperitoneally (i.p.) to the mice. After 30 min, pentobarbital (30 mg/kg, i.p.) was injected to induce sleep. Flumazenil (2 mg/kg) was injected 30 min before diazepam or HAE. The animals were considered asleep if they stayed motionless when positioned on their back and also when they lost their righting reflex. The time interval between injection of pentobarbital and start of sleep was considered as sleep latency.

**Determination of LD50**

For determination of LD50 of HAE, nine groups of 2 mice were used. Groups 1-8 received 50, 100, 200, 400, 800, 1600 and 3200 mg/kg of HAE (i.p.) and group 9 received normal saline (i.p.). Mortality rate was recorded after 24 hr. The maximum dose which did not kill any mice and the minimum dose that led to death of one animal were recorded. The average of these two doses was considered as the median lethal dose (Oliaee et al., 2014 ▶ and Akhila et al., 2007 ▶).

**Rota-rod test**

The rota-rod test was used to measure motor resistance and coordination. The experimental procedure for learning and adaptation was done in 3 consecutive days. On the next day, rats were placed on a rotating rod that accelerated smoothly from 4 to 40 rpm over a period of 5 min. The length of time they could maintain their balance on the turntable against the movement's strength was recorded. Then, the extract or vehicle was injected and after 30 min, the animals were placed on the rota-rod again (Pritchett and Mulder, 2003 ▶; Vafaee et al., 2014 ▶).

**Neurotoxicity valuation**

The rat pheochromocytoma-derived cells (PC12) were cultured in 96-well plates for 24 hr in DMEM supplemented with 10% FBS, penicillin (100 U/ml) and streptomycin (100 µg/ml). Then, the culture medium was changed to new one that contains vehicle (DMSO 1%) or HAE (100, 200, 400 and 800µg/ml). After that, the cells were incubated for 24 hr at 37 ˚C and 5% CO_2_. Then, cell proliferation was evaluated with MTT assay (Hosseini et al., 2014 ▶). Briefly, the MTT solution was added to culture medium to make a final concentration of 0.5 mg/ml and cells were incubated for 2 hr. Then, the medium was discarded and the resulting formazan was dissolved in DMSO. The optical density of dye was measured at 545 nm. The test was done in triplicate and repeated twice. Level of cytotoxicity was expressed as the percentage of living cells.

**Statistical analyses**

All values were expressed as mean ± SEM. Statistical analysis was carried out using one way analysis of variance (ANOVA) followed by Tamhane’s T2 *post-hoc* test. Differences of p<0.05 were considered to be statistically signiﬁcant.

## Results

**HAE increased the duration of sleep**

**Figure 1:**
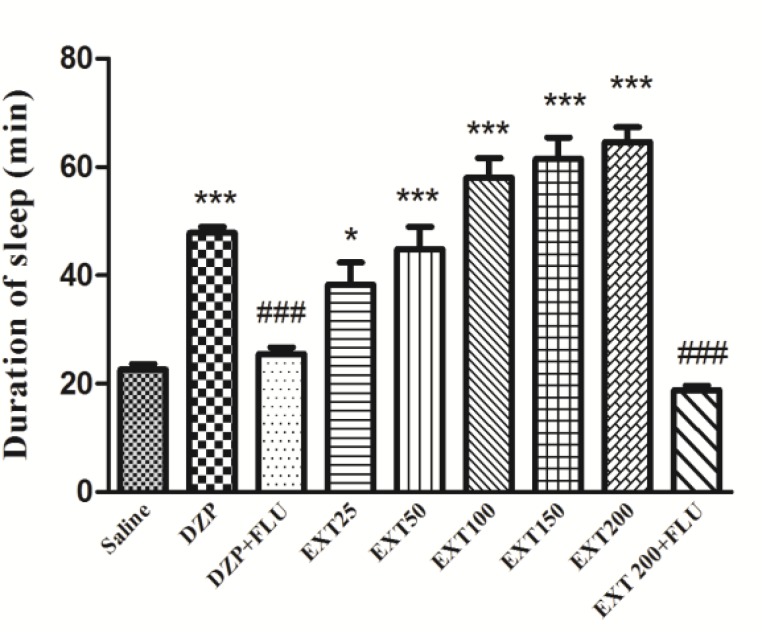
Effects of *Perovskia abrotanoides* hydro-alcoholic extract on sleeping time in pentobarbital-induced hypnotic test. Saline, diazepam (3 mg/kg) and different doses (25, 50, 100, 150 and 200 mg/kg) of the extract were intra-peritoneally administered 30 min before challenging animals with pentobarbital (30 mg/kg, i.p.). Flumazenil (2 mg/kg) was used 30 min before the injection of the extract or diazepam. Data are expressed as mean± SEM of 6-8 animals in each group. *p<0.05 and ***p<0.001 significantly different from control. ^###^p<0.001 significantly different from the EXT plus flumazenil with EXT alone (2mg/kg). DZP: diazepam; EXT: extract; FLU: flumazenil

Sleep duration in sham-treated group was 20.0 ± 0.95 min (Figure 1). As expected, the reference drug diazepam was able to increase the duration of sleep (47.83 ± 1.14 min, p<0.001 *vs.* saline control). The HAE at the doses of 25, 50, 100, 150 and 200 mg/kg significantly increased the sleep duration to 38.33 ± 4.05 min (p<0.05), 44.83± 4.13 min (p<0.001), 58.00± 3.60 min (p<0.001), 61.50 ± 3.96 min (p<0.001), and 64.67± 2.73, respectively. Pretreatment of mice with flumazenil decreased the sleep-prolonging effect of diazepam (47.83 ± 1.138 and 25.50±1.17 min, respectively) (p<0.001). Similarly, the effect of HAE on the sleep duration was significantly inhibited by flumazenil (64.67 ± 2.728 and 18.83 ± 0.7923 min, respectively) (p<0.001) (Figure 1).

**Effect of HAE on sleep latency**

**Figure 2:**
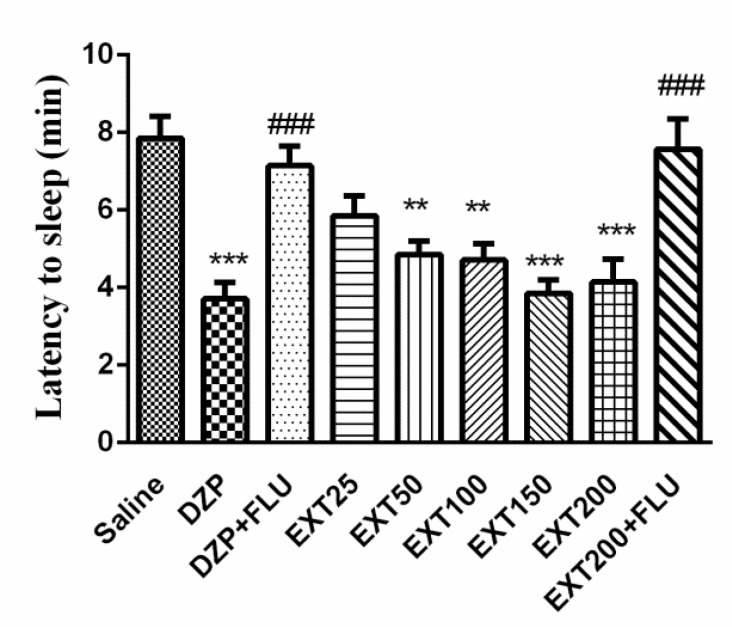
Effects of *Perovskia abrotanoides* hydro-alcoholic extract on sleeping latency in pentobarbital-induced hypnotic test. Saline, diazepam (3 mg/kg) and different doses (25, 50, 100, 150 and 200 mg/kg) of the extract were intraperitoneally administered 30 min before challenging animals with pentobarbital (30 mg/kg, i.p.). Flumazenil (2 mg/kg) was used 30 min before the injection of the extract or diazepam. Data are mean ± SEM of 6-8 animals in each group. ***p<0.001 and **p<0.01 significantly different from control group. ###p<0.001 significantly different from the EXT plus flumazenil with EXT alone (2 mg/kg). DZP: diazepam; EXT: extract; FLU: flumazenil

The sleep latency in saline group was 7.86 ± 0.55 min. Diazepam (3.71 ± 0.42 min, p<0.001) and HAE at doses of 50 (4.86 ±0.34 min, p<0.01), 100 (4.71 ± 0.42 min, p<0.01), 150 (3.85 ± 0.34 min, p<0.001) and 200 mg/kg (4.14 ± 0.59 min, p<0.001) significantly reduced the sleep latency. As it can be observed in Figure 2, flumazenil (2 mg/kg, i.p.) reversed the effects of diazepam (7.14 ±0.51 *vs* 3.71 ± 0.42 min, respectively) (p<0.001) and the effects of HAE 200 mg/kg (7.57 ± 0.78 *vs* 4.14 ± 0.59) (p<0.001).

**Effect of HAE fractions on sleep duration**

**Figure 3:**
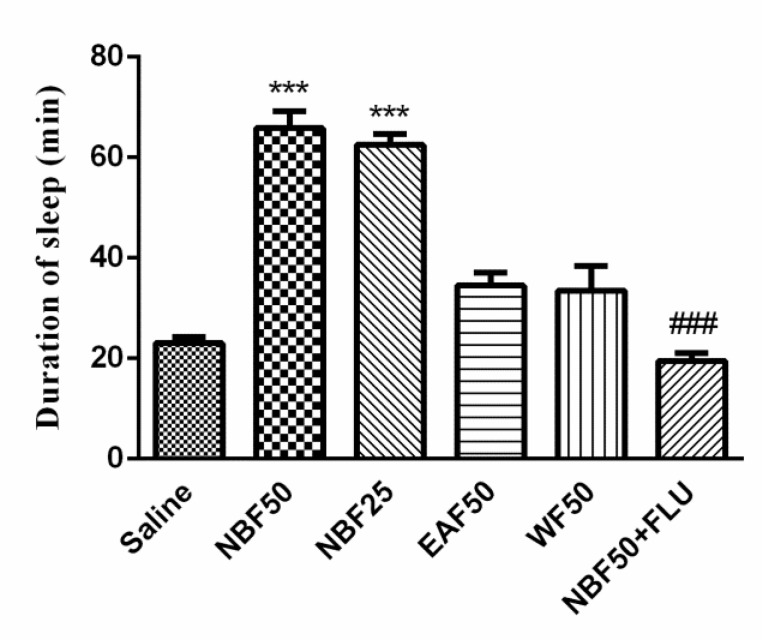
Effects of hydro-alcoholic extract fractions of *Perovskia abrotanoides* on sleeping time in pentobarbital-induced hypnotic test. The WF, EAF and NBF were intraperitoneally administered 30 min before challenging animals with pentobarbital (30mg/kg, i.p.). Data are mean±SEM. of 6-8 animals in each group. ***p<0.001 significantly different from control. ^###^p<0.001 significantly different from the BNF plus flumazenil with NBF alone (2 mg/kg). DZP: diazepam; FLU: flumazenil; WF: water fraction, EAF: ethyl acetate fraction and NBF: n-butanol fraction

The sleep duration in saline group was 23.00±1.26 min. Only NBF at the doses of 25 mg/kg (62.50±2.14 min) and 50 mg/kg (65.83±3.36 min, p<0.001) was able to significantly increase the sleep time. However, WF and EAF effects were statistically non-significant. Also, flumazenil reversed the effects of NBF (19.5±1.56 *vs *65.83±3.361 min) (p<0.001) (Figure 3).

**Effect of HAE fractions on sleep latency**

**Figure 4:**
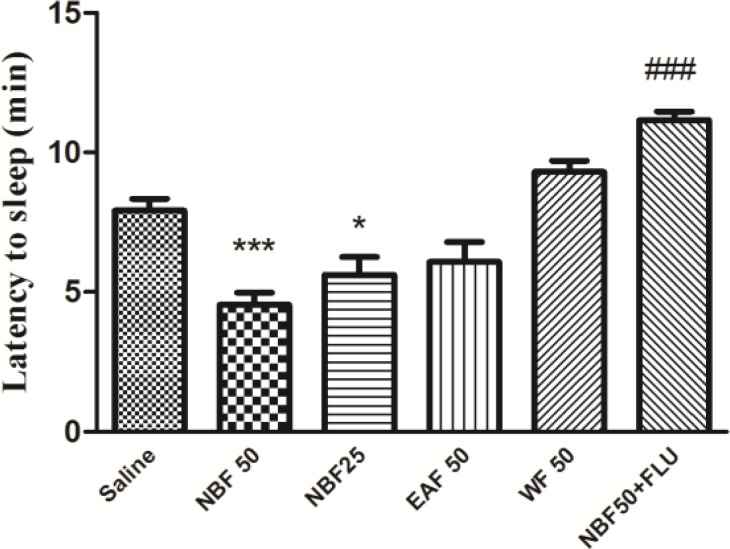
Effects of hydro-alcoholic extract fractions of *Perovskia abrotanoides* on sleeping latency in pentobarbital-induced hypnotic test. The WF, EAF and NBF were intraperitoneally administered 30 min before challenging animals with pentobarbital (30 mg/kg, i.p.). Data are mean ± SEM. of 6-8 animals in each group. ***p<0.001 and *p<0.05 significantly different from control. ^###^p<0.001 significantly different from the NBF plus flumazenil with NBF alone (2 mg/kg). DZP: diazepam; FLU: flumazenil; WF: water fraction, EAF: ethyl acetate fraction and NBF: n-butanol fraction

The sleep latency in saline group was 7.92 ± 0.415. NBF at the doses of 25 mg/kg (5.61± 0.64, p<0.05) and 50 mg/kg (4.53±0.44, p<0.001) significantly reduced the sleep latency. Flumazenil significantly reversed the effect of NBF on the sleep latency (11.15± 0.32*vs *4.54 ± 0.45, p<0.001) (Figure 4).

**Toxicity assessments**

The highest dose of HAE which did not kill any mice and the lowest dose which led to death of one mouse were 6.4 and 3.2 g/kg, respectively. The mean of these two doses (4.8 g/kg) was considered as LD50.

**Figure 5:**
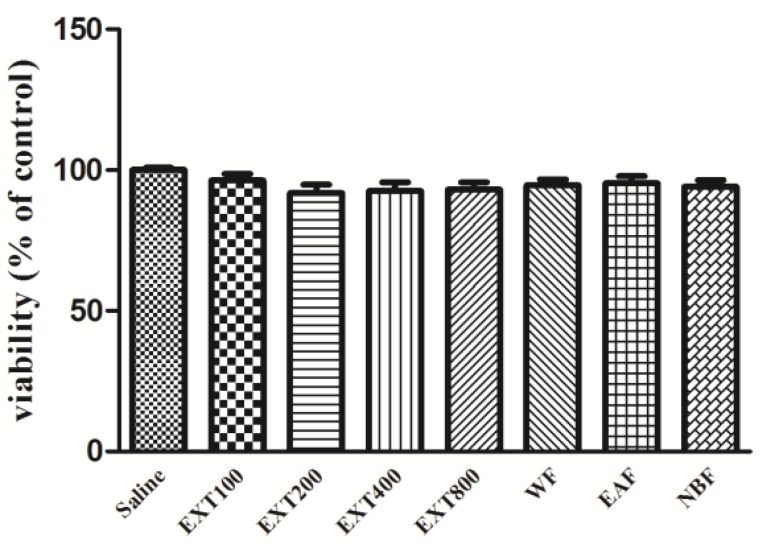
Effect of hydro-alcoholic extract of *Perovskia abrotanoides* and its fractions on PC12 cell viability. Cell viability was quantitated by MTT assay. PC12 cells were exposed to extract for 24 hr. Results are mean ± SEM. EXT: extract

Results showed that none of the HAE concentrations decreased the proliferation of PC12 cells. In the presence of 100, 200, 400 and 800 µg/ml of the extract, survival of the cells was 96.25± 2.39, 91.75± 3.12, 92.50±3.23 and 93.00±2.65%, respectively, as compared to control. Similarly, the HAE fractions exhibited no cytotoxicity. The level of viability was 94.50 ± 2.21, 95.25 ± 2.56 and 94.00 ± 2.45 for WF, EAF and NBF, respectively (Figure 5).

In the rota-rod performance, the statistical analysis revealed that the latency of fall did not decrease in rats receiving 50 mg/kg HAE. The animals of the control group maintained their balance on the rotating rod for 42.0 ± 8.0 sec.

## Discussion

Nowadays insomnia is a common problem worldwide. It influences individuals’ physical and mental status and patients use hypnotic drugs to initiate and maintain the sleep (Orzel-Gryglewska, 2010 ▶; Zaharna and Guilleminault, 2010 ▶). Unfortunately, long term consumption of these drugs leads to different side effects such as tolerance and dependence (Valli`eres et al., 2005 ▶). In traditional medicine, some plants are known to have sedative effects. However, researchers attempt to use these plants for treatment of diseases such as insomnia. These compounds alone or with hypnotic drugs may be effective in reducing side effects or potentiating the therapeutic effects of synthetic drugs. One of these herbs is *Perovskia abrotanoides*. It has been used in traditional medicine as a sedative, analgesic and antiseptic agent (Moallem and Niapour, 2008 ▶; Hosseinzade et al., 2001 ▶; Nassiri et al., 2000 ▶). In this research, we investigated the hypnotic effects of *P. abrotanoides* for the first time. Our results showed that hydro-alcoholic extract and the n-butanol fraction increased the hypnotic effect of pentobarbital and decreased sleep latency. Also, neurotoxicity test revealed that it did affect the cell viability. Because HAE did not affect the animals’ performance on the rota-rod test, we assume that its effects on sleeping time and sleep latency, are not due to the effects on motor movement.

Diazepam as a benzodiazepine drug acts via GABA receptor (GABA-A) (Huang et al., 2007 ▶). Past studies have shown that GABA receptors play an important role in latency and duration of sleep (Herrera-Ruiz et al., 2007 ▶). HAE administration at the doses of 25-200 mg/kg produced sedative effect similar to that observed with 3 mg/kg of diazepam. Since HAE had similar effect of diazepam, it is proposed that HAE probably acts via GABAergic system. The inhibitory action of GABA is mediated via opening of chloride channels and hyperpolarization of the membrane, all leading to CNS depression, and sedative and hypnotic activity (Alnamer et al., 2012 ▶). Therefore, the drugs that influence these systems can be beneficial in the treatment of insomnia disorder. In this research, the potentiating effect of HAE on sleep was observed. It not only prolonged the sleeping time, but also decreased the latency of falling asleep. In order to determine if benzodiazepine receptors participate in the hypnotic effects of HAE, flumazenil as a specific antagonist of the benzodiazepine receptors was administered. Pre-treatment with flumazenil significantly reduced the effects of HAE. Therefore, it is possible that the HAE increases sleep via benzodiazepine receptor.

In this research, in order to determine the effective compounds of HAE, three fractions were prepared: (1) The WF solubilizing which contains polar agents and water-soluble constituents of the plant (e.g. glycosides, quaternary alkaloids and tannins); (2) the EAF extracting which contains compounds with intermediate polarity; and (3) the NBF that has non-polar agents like sterols, alkanes and some terpenoids (Ghorbani et al., 2012 ▶). The present data showed that NBF was the only fraction which could significantly prolong the sleep duration or decrease the sleep latency. Also, flumazenil reversed NBF effect on sleep duration. The phytochemical screening of the aerial parts of *P. abrotanoides* has shown the presence of high content of monoterpenes and sesquiterpenes like 1, 8-cineolo, myrcene, pinene, camphor, caryophyllene, humulene, camphene and bisabolol (Morteza-Semnani 2004 ▶; Sajjadi et al., 2005 ▶). The root of this plant contains diterpenoides such as miltirone (Sairafianpour et al., 2001 ▶). Also, diterpenes might be present in the aerial parts of *P. abrotanoides*. Past studies have shown miltirone as a centrally-acting benzodiazepine receptor partial agonist has tranquilizing effect. Therefore, the observed effects of *P. abrotanoides* may be related to the presence of miltirone (Lee et al., 1991 ▶). Also, Shah et al. (2013) ▶ showed that *P. abrotanoides *extract has a spasmolytic activity via blocking calcium which can induce sleep in mice (Shah et al., 2013 ▶). Recent studies have shown that classical calcium channel blockers, like nifedipine, verapamil and diltiazem prolong sleeping time in mice treated with the pentobarbital (Zhao et al., 2006 ▶) which support our findings. The toxicity assay showed that LD50 value for HAE of *P. abrotanoides* is 4.8 g/kg. This dose is much higher that the hypnotic doses (25-200 mg/kg). Also, HAE even at high concentrations did not decrease the viability of neuronal cells. Therefore, it seems that hypnotic effect of *P. abrotanoides* is accompanied with no neurotoxicity. In conclusion, this work showed that *P. abrotanoides *has significant sedative-hypnotic effect. Further chemical and pharmacological analyses of the extract are needed to isolate and characterize the active components responsible for the sedative effect of *P. abrotanoides*.
